# Impact of thoracic shape on the surgical outcomes of laparoscopic‐assisted living donor hepatectomy

**DOI:** 10.1002/ags3.12755

**Published:** 2023-11-17

**Authors:** Kosuke Tanaka, Satoshi Ogiso, Tomoaki Yoh, Ahmed Hussein Abdelhafez, Yuki Masano, Shinya Okumura, Shoichi Kageyama, Takashi Ito, Koichiro Hata, Etsuro Hatano

**Affiliations:** ^1^ Department of Surgery, Graduate School of Medicine Kyoto University Kyoto Japan; ^2^ Department of General Surgery Ain Shams University Cairo Egypt

**Keywords:** donor hepatectomy, laparoscopic hepatectomy, laparoscopy, liver transplantation, thoracic shape

## Abstract

**Background:**

Although laparoscopic‐assisted donor hepatectomy (LADH) has become the definitive procedure for harvesting living donor livers, its surgical outcomes in association with donor body shape have not been elucidated.

**Methods:**

The impact of donor factors, including thoracic shape, on LADH outcomes was retrospectively investigated. Thoracic anthropometric data were examined in all LADHs with a left/right graft between 2013 and 2022.

**Results:**

The study included 210 LADHs, consisting of 106 left‐ and 104 right‐lobe donors with similar blood loss and similar operation time. Males have greater thoracic depth and greater thoracic width compared with females, respectively. Thoracic depth was associated with graft weight (*p* < 0.001), blood loss (*p* < 0.001), and operation time (*p* < 0.001). On multivariate analyses, blood loss >500 mL and operation time >8 h were associated with graft weight in the left‐lobe donors, and blood loss >500 mL was associated with thoracic depth in the right‐lobe donors.

**Conclusion:**

The greater thoracic depth is associated with massive blood loss in right‐lobe donors. Anthropometric parameters might be helpful for estimating LADH outcomes.

## Graphical abstract

A greater thoracic depth contributes to blood loss >500 mL in right‐lobe donors. This anthropometric parameter is helpful for estimating LADH outcomes and should be considered in preoperative planning to ensure the highest safety of living donor hepatectomy.

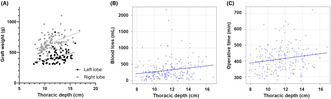

## 1 INTRODUCTION

Liver transplantation is a treatment for end‐stage liver disease and liver failure, and due to the scarcity of deceased donor liver grafts, living donor liver transplantation (LDLT) has become widely accepted as a valid option.^1^, ^2^ LDLT donors are healthy without any liver disease, but donor hepatectomy is still demanding, requiring both special attention to the graft liver quality and the donor's remnant liver. Since the first report of laparoscopic left lateral graft procurement for infants in 2002,^3^ minimally invasive donor hepatectomy (MIDH) has increased in popularity worldwide, and its indication has expanded from left lateral graft procurement in pediatric LDLT^4^, ^5^ to left and right graft procurement in adult LDLT.^6^, ^7^, ^8^, ^9^, ^10^ Laparoscopic‐assisted donor hepatectomy (LADH) is currently used in 50.8%–57.5% of MIDH with the right and left graft, as reported in an international multicenter study^6^ and a world survey.^11^

The donor safety is paramount during the living procurement,^5^, ^12^ factors associated with LADH outcomes should be thoroughly investigated. Although the advantages of MIDH, such as better cosmesis, reduced pain, and shorter hospital stay, can reduce the physical and psychological burden on donors,^13^ limiting the length of skin incision during LADH can make the procedure more demanding and compromise donor outcomes, especially in the deep operative field in thick abdomen. However, impacts of thoracic anthropometric parameters on LADH outcomes have not been evaluated yet.

The aim of this study was to evaluate factors associated with LADH outcomes, with special reference to anthropometric parameters^14^, ^15^, ^16^ of the donors' thoracic shape.

## 2 MATERIALS AND METHODS

### 2.1 Study design

From a prospectively maintained institutional database on liver transplantation, 312 LDLTs performed between August 2013 and April 2022 were identified. Among them, 210 patients who underwent MIDH for a right or left graft were included in the study, while 91 patients who received left lateral, right posterior, or monosegment grafts and 11 patients who underwent open donor hepatectomy were excluded from the study. All donor hepatectomies were performed using a laparoscopic‐assisted hybrid approach. Donor variables, such as age, body mass index (BMI), and anthropometric parameters on the thorax, as well as donor surgical data, were retrieved from the patients' medical records. We evaluated the association between anthropometric parameters and LADH surgical outcomes, including blood loss, surgery time, and major complications. Major complications were defined as conversion cases to conventional open surgery and complications with a Clavien–Dindo Grade ≥3.^17^ Postoperative bile leakage was classified in accordance with the International Study Group for Liver Surgery's definition.^18^ Liver‐to‐spleen CT attenuation values ratio (L/S ratio) were adopted to assess the degree of steatosis. The L/S ratio was calculated based on the previous report.^19^ Surgeon experience (trainees versus experts) and institutional learning curve (the former versus latter period) was also analyzed as confounders. This study conformed to the principles outlined in the ethical guidelines of the World Medical Association Declaration of Helsinki 2013 and the Declaration of Istanbul 2018. The study was approved by the Kyoto University ethics committee (R1473‐3), and the requirement for written informed consent was waived due to the retrospective nature of this study.

### 2.2 Laparoscopic‐assisted donor hepatectomy

At Kyoto University Hospital, LADH has been the primary choice of technique for donor hepatectomy since 2013. The liver graft was chosen to have an estimated graft‐to‐recipient weight ratio (GRWR) ≥0.6% and to preserve a remnant liver volume ≥30%.^20^ Technical details were described previously.^21^ Briefly, two 5‐mm trocars are inserted into the right upper abdomen and the umbilicus. An assistant inserted their hand through an 8‐cm upper midline incision to retract the liver. The right liver was mobilized using the hand‐assisted laparoscopic approach, and several short hepatic veins were ligated and divided as needed. The midline incision was extended to 12–14 cm to allow hilar dissection and parenchymal transection using the hybrid approach. Liver parenchyma was transected under direct vision using a Cavitron ultrasonic surgical aspirator. The hanging maneuver was routinely used during the parenchymal transection. The Pringle maneuver, an inflow occlusion technique, was not used. Intraoperative cholangiography was routinely performed to identify the best location for bile duct division and to prevent biliary stenosis. Abdominal drainage was not placed on principle.

### 2.3 Anthropometric data on thoracic shape

**FIGURE 1:**
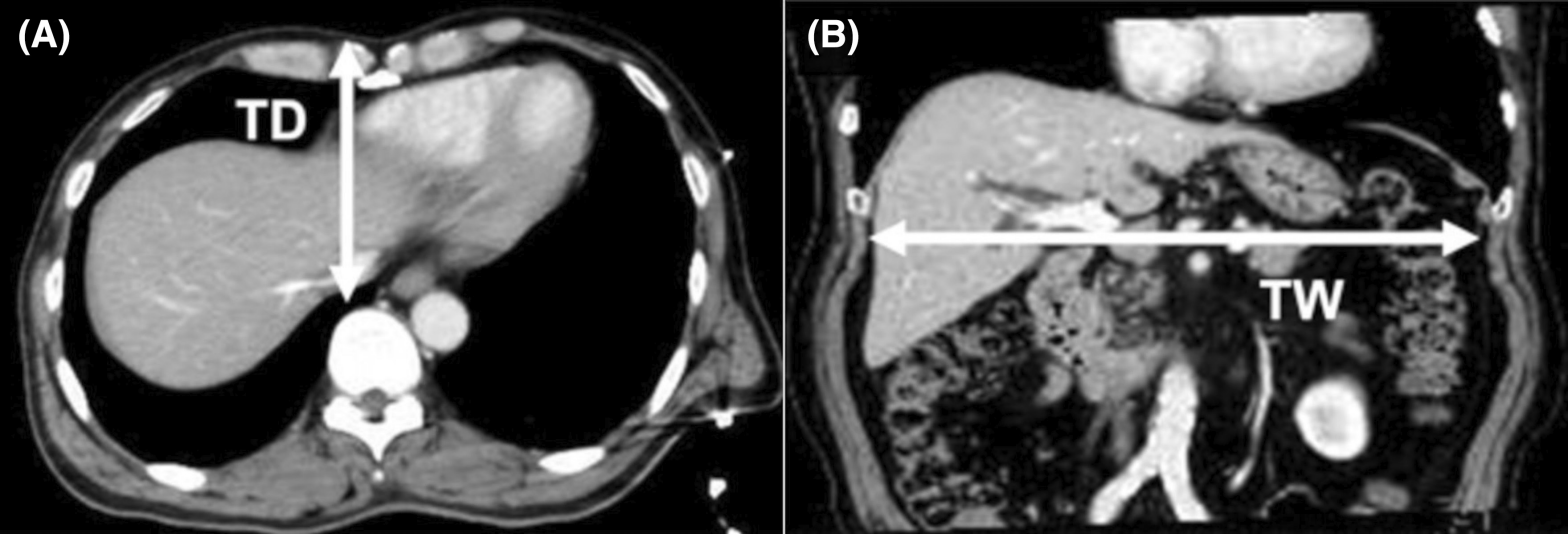
Illustration of measurements of thoracic anthropometric parameters. (A) thoracic depth (TD). (B) thoracic width (TW).

Thoracic morphology was evaluated using pooled contrast‐enhanced computed tomography data (Figure 1). Thoracic depth was defined as the distance between the skin and the anterior surface of the vertebra at the level of the suprahepatic inferior vena cava. Thoracic width was defined as the width of the bilateral ribs at their widest point in space.

### 2.4 Statistical analysis

All statistical analyses were performed using GraphPad Prism (Version 9.3.1, GraphPad Software). Categorical data were compared using Fisher's exact test. Continuous data were presented as the median and interquartile range and were compared using the Mann–Whitney *U* test. Simple linear regression analysis was performed using a scatterplot between each factor, and the correlation coefficient (*r*) was calculated using Pearson's correlation test. The odds ratio (OR) of factors impacting the surgical outcome was analyzed using logistic regression analysis and described using the 95% confidence interval (CI). Variables were selected for multivariate analysis by BIC (Bayesian Information Criterion) in a stepwise algorithm. All *p*‐values were two‐sided, and *p* < 0.05 was considered to be statistically significant.

## 3 RESULTS

### 3.1 Thoracic anthropometric data of male versus female

Thoracic depth and width of male and female donors were summarized in Table 1, together with other demographics. Males had significantly higher BMI, greater thoracic depth, and greater thoracic width, compared with females, respectively.

**TABLE 1:** Differences in thoracic anthropometric parameters between male and female donors.

	Male (*n* = 92)	Female (*n* = 118)	*p* Value
Age, years	38 [30, 53]	46 [35, 56]	<0.01
BMI, kg/m^2^	22.6 [21.0, 24.6]	21.3 [19.4, 23.2]	<0.001
L/S ratio	1.21 [1.17, 1.32]	1.27 [1.18, 1.35]	0.08
Thoracic depth, cm	12.7 [11.5, 13.8]	10.4 [9.4, 11.6]	<0.001
Thoracic width, cm	25.8 [24.7, 27.3]	22.7 [21.8, 23.9]	<0.001

Abbreviations: BMI, body mass index; L/S ratio, liver‐to‐spleen computed tomography attenuation values ratio.

### 3.2 Donor characteristics and surgical outcomes

**FIGURE 2:**
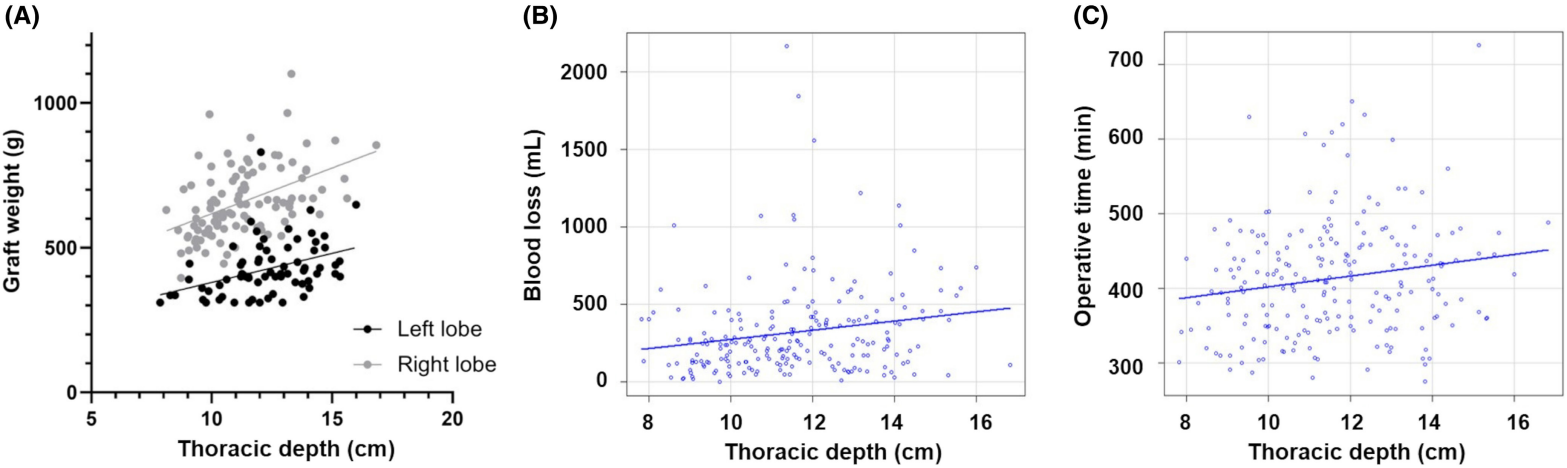
Plotting of graft weight (A), blood loss (B), and operation time (C) in association with thoracic depth.

Donor background characteristics and surgical outcomes were summarized in Table 2, comprising 106 left (50.4%) and 104 right (49.6%) grafts. The left lobe donors retained the Spiegel lobe (the left part of segment 1) in the donor's remnant liver, except for one case in which the left lobe graft was combined with the Spiegel lobe. The proportion of females was significantly higher and thoracic depth and thoracic width were both greater in left‐lobe donor, respectively. Graft weight was significantly less in the left‐lobe donors compared with right‐lobe donors (Table 2), and associated with thoracic depth in both of left‐ (*r* = 0.39, *p* < 0.001) and right‐lobe donors (*i* = 0.47, *p* < 0.001), respectively (Figure 2A). Blood loss and operation time were similar between left‐ and right‐lobe donors, respectively (Table 2), while blood loss and operation time were associated with thoracic depth, respectively (Figure 2B,C; blood loss, *r* = 0.19, *p* = 0.007; operation time, *r* = 0.18, *p* = 0.01). Conversion to conventional open surgery, which occurred in four patients due to significant blood loss or an inadequate field of view, was similar between left‐ and right‐lobe donors. Postoperative biliary leakage and major complications, classified as Clavien–Dindo Grade IIIa in five and Clavien–Dindo Grade IIIb in three, were also similar between left‐ and right‐lobe donors. Reoperations were indicated due to upper gastrointestinal perforation, liver subcapsular hematoma, and a major bile leak.

**TABLE 2:** Background characteristics and surgical outcomes.

	Left‐lobe donor (*n* = 106)	Right‐lobe donor (*n* = 104)	Total (*n* = 210)	*p* Value
Sex, male (%)	61 (57.5)	31 (29.8)	92 (43.8)	<0.001
Age, years	38 [30, 48]	51 [37, 57]	43 [32, 55]	<0.001
BMI, kg/m^2^	22.1 [20.6, 24.4]	21.7 [19.9, 23.4]	21.9 [20.3, 23.8]	0.12
L/S ratio	1.24 [1.17, 1.34]	1.23 [1.18, 1.32]	1.23 [1.17, 1.33]	0.7
Previous abdominal surgery (%)	9 (8.5)	8 (7.7)	17 (8.1)	1
Thoracic depth, cm	11.9 [10.4, 13.2]	11.0 [10.0, 12.6]	11.4 [10.0, 13.0]	0.02
Thoracic width, cm	24.7 [23.0, 26.2]	23.6 [21.9, 25.1]	24.0 [22.4, 25.6]	0.001
Graft weight, g	393 [330, 440]	650 [565, 732]	518 [391, 650]	<0.001
Blood loss, g	245 [131, 407]	248 [124, 404]	245 [129, 407]	0.78
Operation time, min	400 [350, 459]	415 [364, 456]	409 [356, 459]	0.39
Surgeon experience, trainee (%)	18 (17.0)	10 (9.6)	28 (13.3)	0.155
Clavien–Dindo classification (%)				
I–II	28 (26.4)	20 (19.2)	48 (22.9)	0.07
IIIa	4 (3.8)	1 (1.0)	5 (2.3)	
IIIb	3 (2.8)	0 (0)	3 (1.4)	
IV	0 (0)	0 (0)	0 (0)	
V	0 (0)	0 (0)	0 (0)	
Conversion (%)	2 (1.9)	2 (1.9)	4 (1.9)	1
Transfusion (%)	0 (0)	1 (1.0)	1 (0.5)	0.5
Biliary leakage (%)	5 (4.7)	1 (1.0)	6 (2.9)	0.21
Abdominal fluid (%)	3 (2.8)	1 (1.0)	4 (1.9)	0.62
Reoperation (%)	3 (2.8)	0 (0)	3 (1.4)	0.25
Hospital stay, days	12 [10, 14]	12 [10, 14]	12 [10, 14]	0.89

*Note*: Continuous values are expressed as median [interquartile range].

Abbreviations: BMI, body mass index; L/S ratio, liver‐to‐spleen computed tomography attenuation values ratio.

### 3.3 Predictive factors associated with unfavorable surgical outcomes

Univariate and multivariate analyses for factors associated with unfavorable surgical outcomes, such as blood loss >500 mL, operation time >8 h, and major complications were summarized in Tables 3, 4, 5. There were 36 cases of blood loss >500 mL and 31 cases of operation time >8 h.

**TABLE 3:** Univariate and multivariate analysis of predictive factors for blood loss >500 mL in left‐lobe donors (A) and right‐lobe donors (B).

	Univariate analysis		Multivariate analysis	
	OR [95% CI]	*p* Value	OR [95% CI]	*p* Value
A. Left‐lobe donors				
Sex (ref. male)	0.47 [0.17, 1.33]	0.16		
Age	1.01 [0.97, 1.05]	0.63		
BMI (kg/m^2^)	1.13 [0.96, 1.34]	0.13		
L/S ratio	10.60 [0.19, 589.00]	0.25		
Graft weight (g)	1.01 [1.00, 1.01]	0.01	1.01 [1.00, 1.01]	0.01
Surgeon experience	2.43 [0.79, 7.51]	0.12		
Institutional learning curve (the latter period)	1.13 [0.43, 2.93]	0.81		
Thoracic depth (cm)	1.33 [1.01, 1.74]	0.04		
Thoracic width (cm)	1.16 [0.94, 1.44]	0.16		
B. Right‐lobe donors				
Sex (ref. male)	0.42 [0.14, 1.29]	0.13		
Age	1.00 [0.96, 1.05]	0.88		
BMI (kg/m^2^)	1.21 [1.00, 1.47]	0.048		
L/S ratio	0.34 [0.00, 51.20]	0.67		
Graft weight (g)	1.00 [1.00, 1.01]	0.33		
Surgeon experience	1.56 [0.30, 8.16]	0.6		
Institutional learning curve (the latter period)	0.62 [0.21, 1.90]	0.41		
Thoracic depth (cm)	1.48 [1.09, 2.01]	0.01	1.47 [1.08, 1.99]	0.01
Thoracic width (cm)	1.18 [0.93, 1.49]	0.18		

Abbreviations: BMI, body mass index; CI, confidence interval; L/S ratio, liver‐to‐spleen computed tomography attenuation values ratio; OR, odds ratio.

**TABLE 4:** Univariate and multivariate analysis of predictive factors for operation time >8 h in left‐lobe donors (A) and right‐lobe donors (B).

	Univariate analysis		Multivariate analysis	
	OR [95% CI]	*p* Value	OR [95% CI]	*p* Value
A. Left‐lobe donors				
Sex (ref. male)	0.24 [0.06, 0.89]	0.03		
Age	1.02 [0.98, 1.07]	0.3		
BMI (kg/m^2^)	1.21 [1.01, 1.45]	0.04		
L/S ratio	6.77 [0.09, 517.00]	0.39		
Graft weight (g)	1.01 [1.00, 1.01]	0.005	1.01 [1.00, 1.01]	0.01
Surgeon experience	0.00 [0.00, –]	0.99		
Institutional learning curve (the latter period)	0.17 [0.04, 0.62]	0.01	0.18 [0.05, 0.70]	0.01
Thoracic depth (cm)	1.28 [0.96, 1.71]	0.09		
Thoracic width (cm)	1.28 [1.01, 1.62]	0.04		
B. Right‐lobe donors				
Sex (ref. male)	0.73 [0.22, 2.39]	0.6		
Age	1.01 [0.97, 1.05]	0.72		
BMI (kg/m^2^)	1.14 [0.94, 1.38]	0.2		
L/S ratio	0.54 [0.00, 88.30]	0.81		
Graft weight (g)	1.00 [0.99, 1.00]	0.99		
Surgeon experience	0.69 [0.08, 5.93]	0.74		
Institutional learning curve (the latter period)	0.23 [0.06, 0.87]	0.03	0.23 [0.06, 0.87]	0.03
Thoracic depth (cm)	1.20 [0.89, 1.63]	0.23		
Thoracic width (cm)	1.18 [0.92, 1.50]	0.19		

Abbreviations: BMI, body mass index; CI, confidence interval; L/S ratio, liver‐to‐spleen computed tomography attenuation values ratio; OR, odds ratio.

**TABLE 5:** Univariate analyses of predictive factors for major complications in left‐lobe donors (A) and right‐lobe donors (B).

	OR [95% CI]	*p* Value
A. Left‐lobe donors		
Sex (ref. male)	0.31 [0.06, 1.53]	0.15
Age	0.99 [0.93, 1.04]	0.62
BMI (kg/m^2^)	0.99 [0.79, 1.24]	0.91
L/S ratio	0.18 [0.00, 51.10]	0.55
Graft weight (g)	1.00 [0.99, 1.01]	0.22
Surgeon experience	0.00 [0.00, –]	0.99
Institutional learning curve (the latter period)	1.0 [0.27, 3.68]	1
Thoracic depth (cm)	1.28 [0.89, 1.84]	0.18
Thoracic width (cm)	0.93 [0.69, 1.26]	0.65
B. Right‐lobe donors		
Sex (ref. male)	N/A	N/A
Age	1.02 [0.93, 1.12]	0.73
BMI (kg/m^2^)	0.93 [0.60, 1.44]	0.76
L/S ratio	176.0 [0.04, 86200.00]	0.23
Graft weight (g)	0.99 [0.98, 1.00]	0.24
Surgeon experience	5.11 [0.42, 62.0]	0.2
Institutional learning curve (the latter period)	2.04 [0.18, 23.20]	0.57
Thoracic depth (cm)	1.00 [0.53, 1.92]	0.99
Thoracic width (cm)	0.79 [0.45, 1.39]	0.42

Abbreviations: BMI, body mass index; CI, confidence interval; L/S ratio, liver‐to‐spleen computed tomography attenuation values ratio; N/A = not applicable; OR, odds ratio.

In the left‐lobe donors, greater graft weight and greater thoracic depth were associated with blood loss >500 mL by univariate analysis, and greater graft weight was an independent predictor of blood loss >500 mL by a multivariate analysis (Table 3). Male, greater BMI, greater graft weight, greater thoracic width, and the former period were associated with operation time >8 h by univariate analysis, and greater graft weight and the former period were independent predictors of operation time >8 h by a multivariate analysis (Table 4). No factors were associated with major complications by a univariate analysis (Table 5).

In the right‐lobe donors, greater BMI and greater thoracic depth were associated with blood loss >500 mL by univariate analysis, and greater thoracic depth was an independent predictor of blood loss >500 mL by multivariate analysis (Table 3). The former period was associated with operation time >8 h by univariate and multivariate analysis (Table 4). No factors were associated with major complications by univariate analysis (Table 5).

## 4 DISCUSSION

Technical challenges are often experienced by liver surgeons during laparoscopic‐assisted major hepatectomy for patients with thick abdomen, typically in heavyset males; however, impacts of body habitus on surgical outcomes have not been fully studied to date. This is the first study to confirm that thoracic shape is associated with technical difficulty of LADH; greater thoracic depth increases blood loss in the right‐lobe donors but does not increase operation time and major complications. These findings help to maximize donor safety and offer the benefits of LADH by selecting the best suitable surgical approach and surgical team based on each donor's thoracic shape.

Predicting the technical difficulty is especially important in donor hepatectomy because donor safety is paramount for a successful LDLT.^22^ Previous studies revealed that overall, up to 20%–33.3% of living donors experienced postoperative morbidity,^6^, ^23^ specifically blood loss (>300 mL) in 23.5% of donors, long surgery time (>400 min) in 51.7%, and bile leakage in 1.3%–6.5% of donors.^24^, ^25^ Similarly, our LADH patients experienced a median blood loss of 245 mL, median operation time of 409 min, and biliary leakage in 2.9% of donors. Vascular anomalies were reported to correlate with donor complications,^24^, ^26^ and the Expert Consensus Guidelines recommended that MIDH for donors with difficult anatomy should only be considered at centers that have well‐established procedures.^13^ A large graft weighing >700 g may also predict increased MIDH difficulty.^13^, ^27^ Similarly, larger grafts were associated with left‐lobe donor outcomes in the current study. Moreover, greater thoracic depth was an independent predictor of blood loss >500 mL in the right‐lobe donors, although a previous study did not reveal the impact of body habitus on blood loss because of its small sample size.^16^

The association between thoracic depth and blood loss in right‐lobe donors is probably attributable to the limited access to the deep surgical field via the small incision^13^ in patients with a deep thorax. Limited exposure disturbs precise parenchymal dissection and meticulous hemostasis, which are directly associated with vascular injury and bleeding. Because the ventral approach is used in LADH,^28^, ^29^, ^30^ the hanging technique is required to improve access to the deep field during LADH,^31^ although our results indicate that using only the hanging technique does not have sufficient safety when performing LADH with a deep thoracic field. One of the solutions would be extending skin incision to ensure safety in case of difficulties.^13^

Marubashi et al. reported previously that another anthropometric parameter, the maximal distance between the surface of the right lobe and the portal vein bifurcation (RPv distance), was significantly associated with operation time in left‐lobe LADH.^32^ RPv distance was strongly associated with TD (left lobe, *r* = 0.40, *p* < 0.01; right lobe, *r* = 0.49, *p* < 0.01) and TW (left lobe, *r* = 0.53, *p* < 0.01; right lobe, *r* = 0.57, *p* < 0.01) as well as graft weight (left lobe, *r* = 0.27, *p* < 0.01; right lobe, *r* = 0.45, *p* < 0.01) in our series, and consequently correlated with blood loss >500 mL in the right‐lobe donors (OR = 2.02 [1.17, 3.48], *p* = 0.01). Thus, the two studies are identical in demonstrating impact of thoracic size on LADH outcomes. Future studies are needed to determine which measurement is best suitable to predict the difficulty of LADH.

Thoracic shape potentially affects the difficulty of other types of hepatectomy, similar to LADH. During open hepatectomy, a deep thorax disrupts access to the dorsal surgical field in the same manner; however, the wide heterogeneity in the complexity and extent of resection have prevented showing the impact of thoracic shape on surgical outcomes. In contrast to LADH with a ventral approach, pure laparoscopic donor hepatectomy (PLDH) is performed using the caudal approach, which offers excellent exposure of the deep surgical field.^28^, ^29^, ^30^ In this manner, PLDH naturally has different predictive factors for its technical difficulty, including anthropometric parameters, and it potentially contributes to better outcomes in donors with a deep thorax. Further research is required to determine the impacts of thoracic shape on PLDH outcomes.

There are some limitations in the present study. This is a single‐institution study for a single ethnicity, which potentially limits the reproducibility of the findings. The retrospective nature of the study requires further prospective studies to confirm the findings.

## 5 CONCLUSION

A greater thoracic depth contributes to greater blood loss in the right‐lobe donors but did not increase the operation time and morbidity. This anthropometric parameter is helpful for estimating LADH outcomes and should be considered in preoperative planning to ensure the highest safety of living donor hepatectomy.

## AUTHOR CONTRIBUTIONS

KT: project development, data analysis & collection, manuscript writing. SO: project development, data analysis & collection, manuscript writing/editing. AHA: project development, data analysis, manuscript editing. TY: project development, data analysis, manuscript editing. YM: project development, data analysis, manuscript editing. SO: project development, data analysis & collection, manuscript editing. SK: project development, data analysis & collection, manuscript editing. TI: project development, data analysis, manuscript editing. KH: project development, data analysis, manuscript editing. EH: project development, data analysis, manuscript editing.

## FUNDING INFORMATION

No funding was received for this work.

## ETHICS STATEMENT

Approval of the research protocol: the study protocol was approved by the institutional review board.

Informed Consent: It was waivered by the institutional review board.

Registry and the Registration No. of the study/trial: NA.

Animal Studies: NA.

## CONFLICT OF INTEREST STATEMENT

The authors declare no conflicts of interest for this article.

## References

[1] Kasiske BL , Lentine KL , Ahn Y , Skeans MA , Eberhard T , Folken C , et al. OPTN/SRTR 2020 annual data report: living donor collective. Am J Transplant. 2022;22(Suppl 2):553–586. 10.1111/ajt.16983 35266611

[2] Lee SG . A complete treatment of adult living donor liver transplantation: a review of surgical technique and current challenges to expand indication of patients. Am J Transplant. 2015;15(1):17–38. 10.1111/ajt.12907 25358749

[3] Cherqui D , Soubrane O , Husson E , Barshasz E , Vignaux O , Ghimouz M , et al. Laparoscopic living donor hepatectomy for liver transplantation in children. Lancet. 2002;359(9304):392–396. 10.1016/s0140-6736(02)07598-0 11844509

[4] Scatton O , Katsanos G , Boillot O , Goumard C , Bernard D , Stenard F , et al. Pure laparoscopic left lateral sectionectomy in living donors: from innovation to development in France. Ann Surg. 2015;261(3):506–512. 10.1097/SLA.0000000000000642 24646560

[5] Kim WJ , Kim KH , Cho HD , Namgoong JM , Hwang S , Park JI , et al. Long‐term safety and efficacy of pure laparoscopic donor hepatectomy in pediatric living donor liver transplantation. Liver Transpl. 2021;27(4):513–524. 10.1002/lt.25910 33021038 PMC8246762

[6] Soubrane O , Eguchi S , Uemoto S , Kwon CHD , Wakabayashi G , Han HS , et al. Minimally invasive donor hepatectomy for adult living donor liver transplantation: an international, multi‐institutional evaluation of safety, efficacy and early outcomes. Ann Surg. 2022;275(1):166–174. 10.1097/sla.0000000000003852 32224747

[7] Soubrane O , Perdigao Cotta F , Scatton O . Pure laparoscopic right hepatectomy in a living donor. Am J Transplant. 2013;13(9):2467–2471. 10.1111/ajt.12361 23865716

[8] Hong SK , Suh KS , Kim KA , Lee JM , Cho JH , Yi NJ , et al. Pure laparoscopic versus open left hepatectomy including the middle hepatic vein for living donor liver transplantation. Liver Transpl. 2020;26(3):370–378. 10.1002/lt.25697 31808294

[9] Eguchi S , Soyama A , Hara T , Natsuda K , Okada S , Hamada T , et al. Standardized hybrid living donor hemihepatectomy in adult‐to‐adult living donor liver transplantation. Liver Transpl. 2018;24(3):363–368. 10.1002/lt.24990 29194959

[10] Suh KS , Hong SK , Lee KW , Yi NJ , Kim HS , Ahn SW , et al. Pure laparoscopic living donor hepatectomy: focus on 55 donors undergoing right hepatectomy. Am J Transplant. 2018;18(2):434–443. 10.1111/ajt.14455 28787763

[11] Rotellar F , Ciria R , Wakabayashi G , Suh KS , Cherqui D , group W‐Mc . World survey on minimally invasive donor hepatectomy: a global snapshot of current practices in 2370 cases. Transplantation. 2022;106(1):96–105. 10.1097/TP.0000000000003680 33586922

[12] Cotter TG , Minhem M , Wang J , Peeraphatdit T , Ayoub F , Pillai A , et al. Living donor liver transplantation in the United States: evolution of frequency, outcomes, center volumes, and factors associated with outcomes. Liver Transpl. 2021;27(7):1019–1031. 10.1002/lt.26029 33619854 PMC9257956

[13] Cherqui D , Ciria R , Kwon CHD , Kim KH , Broering D , Wakabayashi G , et al. Expert consensus guidelines on minimally invasive donor hepatectomy for living donor liver transplantation from innovation to implementation: a joint initiative from the international laparoscopic liver society (ILLS) and the Asian‐Pacific Hepato‐Pancreato‐biliary association (A‐PHPBA). Ann Surg. 2021;273(1):96–108. 10.1097/sla.0000000000004475 33332874

[14] Ratti F , D'Alessandro V , Cipriani F , Giannone F , Catena M , Aldrighetti L . Influence of body habitus on feasibility and outcome of laparoscopic liver resections: a prospective study. J Hepatobiliary Pancreat Sci. 2016;23(6):373–381. 10.1002/jhbp.350 27037539

[15] Russolillo N , Casella M , Langella S , Lo Tesoriere R , Ossola P , Ferrero A . Correlation between anthropometric data and preparatory maneuvers difficulties during laparoscopic right liver resections: a single center prospective study. Surg Endosc. 2022;36:7343–7351. 10.1007/s00464-022-09130-z 35211801

[16] Safwan M , Nagai S , Collins K , Rizzari M , Yoshida A , Abouljoud M . Impact of abdominal shape on living liver donor outcomes in mini‐incision right hepatic lobectomy: comparison among 3 techniques. Liver Transpl. 2018;24(4):516–527. 10.1002/lt.25001 29281863

[17] Dindo D , Demartines N , Clavien PA . Classification of surgical complications: a new proposal with evaluation in a cohort of 6336 patients and results of a survey. Ann Surg. 2004;240(2):205–213. 10.1097/01.sla.0000133083.54934.ae 15273542 PMC1360123

[18] Koch M , Garden OJ , Padbury R , Rahbari NN , Adam R , Capussotti L , et al. Bile leakage after hepatobiliary and pancreatic surgery: a definition and grading of severity by the international study Group of Liver Surgery. Surgery. 2011;149(5):680–688. 10.1016/j.surg.2010.12.002 21316725

[19] Iwasaki M , Takada Y , Hayashi M , Minamiguchi S , Haga H , Maetani Y , et al. Noninvasive evaluation of graft steatosis in living donor liver transplantation. Transplantation. 2004;78(10):1501–1505. 10.1097/01.tp.0000140499.23683.0d 15599315

[20] Kusakabe J , Yagi S , Sasaki K , Uozumi R , Abe H , Okamura Y , et al. Is 0.6% reasonable as the minimum requirement of the graft‐to‐recipient weight ratio regardless of lobe selection in adult living‐donor liver transplantation? Transplantation. 2021;105(9):2007–2017. 10.1097/tp.0000000000003472 33031228

[21] Kitajima T , Kaido T , Iida T , Seo S , Taura K , Fujimoto Y , et al. Short‐term outcomes of laparoscopy‐assisted hybrid living donor hepatectomy: a comparison with the conventional open procedure. Surg Endosc. 2017;31(12):5101–5110. 10.1007/s00464-017-5575-0 28444493

[22] Wan P , Yu X , Xia Q . Operative outcomes of adult living donor liver transplantation and deceased donor liver transplantation: a systematic review and meta‐analysis. Liver Transpl. 2014;20(4):425–436. 10.1002/lt.23836 24478109

[23] Vargas PA , McCracken EKE , Mallawaarachchi I , Ratcliffe SJ , Argo C , Pelletier S , et al. Donor morbidity is equivalent between right and left hepatectomy for living liver donation: a meta‐analysis. Liver Transpl. 2021;27(10):1412–1423. 10.1002/lt.26183 34053171

[24] Lauterio A , Di Sandro S , Gruttadauria S , Spada M , Di Benedetto F , Baccarani U , et al. Donor safety in living donor liver donation: an Italian multicenter survey. Liver Transpl. 2017;23(2):184–193. 10.1002/lt.24651 27712040

[25] Lee JG , Lee KW , Kwon CHD , Chu CW , Kim BW , Choi DL , et al. Donor safety in living donor liver transplantation: the Korean organ transplantation registry study. Liver Transpl. 2017;23(8):999–1006. 10.1002/lt.24778 28431203

[26] Kwon CHD , Choi GS , Kim JM , Cho CW , Rhu J , Soo Kim G , et al. Laparoscopic donor hepatectomy for adult living donor liver transplantation recipients. Liver Transpl. 2018;24(11):1545–1553. 10.1002/lt.25307 30021060

[27] Kim KH , Kang SH , Jung DH , Yoon YI , Kim WJ , Shin MH , et al. Initial outcomes of pure laparoscopic living donor right hepatectomy in an experienced adult living donor liver transplant center. Transplantation. 2017;101(5):1106–1110. 10.1097/TP.0000000000001637 28072754

[28] Soubrane O , Schwarz L , Cauchy F , Perotto LO , Brustia R , Bernard D , et al. A conceptual technique for laparoscopic right hepatectomy based on facts and oncologic principles: the caudal approach. Ann Surg. 2015;261(6):1226–1231. 10.1097/SLA.0000000000000737 24854453

[29] Wakabayashi G , Cherqui D , Geller DA , Han HS , Kaneko H , Buell JF . Laparoscopic hepatectomy is theoretically better than open hepatectomy: preparing for the 2nd international consensus conference on laparoscopic liver resection. J Hepatobiliary Pancreat Sci. 2014;21(10):723–731. 10.1002/jhbp.139 25130985

[30] Ogiso S , Nomi T , Araki K , Conrad C , Hatano E , Uemoto S , et al. Laparoscopy‐specific surgical concepts for hepatectomy based on the laparoscopic caudal view: a key to reboot Surgeons' minds. Ann Surg Oncol. 2015;22(Suppl 3):S327–S333. 10.1245/s10434-015-4661-6 26065871

[31] Nitta H , Sasaki A , Fujita T , Itabashi H , Hoshikawa K , Takahara T , et al. Laparoscopy‐assisted major liver resections employing a hanging technique: the original procedure. Ann Surg. 2010;251(3):450–453. 10.1097/SLA.0b013e3181cf87da 20083994

[32] Marubashi S , Wada H , Kawamoto K , Kobayashi S , Eguchi H , Doki Y , et al. Laparoscopy‐assisted hybrid left‐side donor hepatectomy. World J Surg. 2013;37(9):2202–2210. 10.1007/s00268-013-2117-3 23736986

